# Expression of EMT-related genes in lymph node metastasis in endometrial cancer: a TCGA-based study

**DOI:** 10.1186/s12957-023-02893-2

**Published:** 2023-02-22

**Authors:** Li He, Wang Junzhu, Li Liwei, Zhao Luyang, Wang Zhiqi

**Affiliations:** 1 Department of Obstetrics and Gynecology, Peking University People’s Hospital, No. 11 Xizhimen South Street, Xicheng District, Beijing, 100044 China; 2 The Big Data and Public Policy Laboratory, School of Government, Peking University, Beijing, China

**Keywords:** Endometrial cancer, Lymph node metastasis, Epithelial-mesenchymal transition, The cancer genome atlas, Pathway, Gene signature, Logistic regression model, Nomogram

## Abstract

**Background:**

Endometrial cancer (EC) with metastasis in pelvic/para-aortic lymph nodes suggests an unsatisfactory prognosis. Nevertheless, there is still rare literature focusing on the role of epithelial-mesenchymal transition (EMT) in lymph node metastasis (LNM) in EC.

**Methods:**

Transcriptional data were derived from the TCGA database. Patients with stage IA–IIIC2 EC were included, constituting the LN-positive and LN-negative groups. To evaluate the extent of EMT, an EMT signature composed of 315 genes was adopted. The EMT-related genes (ERGs) were obtained from the dbEMT2 database, and the differentially expressed ERGs (DEERGs) between these two groups were screened. On the basis of DEERGs, pathway analysis was carried out. We eventually adopted the logistic regression model to build an ERG-based gene signature with predictive value for LNM in EC.

**Results:**

A total of 498 patients were included, with 75 in the LN-positive group. Median EMT score of tumor tissues from LN-negative group was − 0.369, while that from the LN-positive group was − 0.296 (*P* < 0.001), which clearly exhibited a more mesenchymal phenotype for LNM cases on the EMT continuum. By comparing expression profiles, 266 genes were identified as DEERGs, in which 184 were upregulated and 82 were downregulated. In pathway analysis, various EMT-related pathways were enriched. DEERGs shared between molecular subtypes were comparatively few. The ROC curve and logistic regression analysis screened 7 genes with the best performance to distinguish between the LN-positive and LN-negative group, i.e., *CIRBP*,* DDR1*,* F2RL2*,* HOXA10*,* PPARGC1A*,* SEMA3E*, and *TGFB1*. A logistic regression model including the 7-gene-based risk score, age, grade, myometrial invasion, and histological subtype was built, with an AUC of 0.850 and a favorite calibration (*P* = 0.074). In the validation dataset composed of 83 EC patients, the model exhibited a satisfactory predictive value and was well-calibrated (*P* = 0.42).

**Conclusion:**

The EMT status and expression of ERGs varied in LNM and non-LNM EC tissues, involving multiple EMT-related signaling pathways. Aside from that, the distribution of DEERGs differed among molecular subtypes. An ERG-based gene signature including 7 DEERGs exhibited a desirable predictive value for LNM in EC, which required further validation based upon clinical specimens in the future.

**Supplementary Information:**

The online version contains supplementary material available at 10.1186/s12957-023-02893-2.

## Background

In 2022, it is expected to be an incidence of 84,520 of uterine corpus cancer in China and 17,543 new deaths, where endometrial cancer (EC) accounts for the vast majority [1]. EC with metastasis in pelvic/para-aortic lymph nodes (LNs), even with micrometastasis, is associated with at least the International Federation of Gynecology and Obstetrics (FIGO) stage IIIC1/2 and suggests a less satisfactory prognosis [2, 3]. LN metastasis (LNM) of cancer is a complex process including lymphangiogenesis and activation of epithelial-mesenchymal transition (EMT) to initiate metastasis and survival in the LN [4–12]. A substantial proportion of studies on EMT in EC have been published [13–17], but literature fixing attention on the role of EMT in LNM remains rare to be reported [18]. As persuasively demonstrated by relevant studies, for primary prevention, adherence to cancer prevention guidelines was negatively associated with EC risk [19, 20]. Nonetheless, for tertiary prevention of EC, the prediction and treatment of LNM remain unsatisfactory. The mechanism-level description of this process will be advantageous for us to facilitate the management of the lymphatic spread of EC.

For this purpose, we presented a retrospective study on the basis of The Cancer Genome Atlas (TCGA) to discuss the role of EMT in LNM, Apart from that, this research not only probed deep into differentially expressed EMT-related genes (DEERGs) and pathways associated with them, but also ultimately provided an ERG-based gene signature with predictive value for LNM in EC.

## Materials and methods

We extracted mRNA expression profiles from the Uterine Corpus Endometrial Carcinoma (TCGA, PanCancer Atlas) database through cBioPortal [21, 22]. Especially, tumor grade and microsatellite status were obtained from Xena [23]. Cases with a non-endometrioid histology were categorized into G3. Patients with FIGO 2009 stage IA–IIIC2 EC were included in this research, and stage IIIC1/2 were considered as stage IA–IIIB with pelvic/para-aortic LNM, constituting two groups (LN-positive and LN-negative). There were cases annotated by FIGO 1988 staging system, and patients with FIGO 1988 stage IIIA were excluded from this study, since peritoneal cytology was contained in this stage, which was absent in FIGO 2009 staging system.

To quantitively locate a tumor tissue on the EMT continuum, a validated transcriptome-based scoring system composed of 315 genes (epithelial: 145, mesenchymal: 170) was adopted. An EMT score was calculated by the maximum vertical distance between the empirical distribution function (ECDF) curves of the mesenchymal and the epithelial gene set by a two-sample Kolmogorov–Smirnov test (i.e., the KS method). The EMT score of a tumor with a higher expression of mesenchymal signatures would be defined as positive, and negative for epithelial ones. Hence, an EMT score in the KS method had a range of [− 1, 1] [24, 25] (Supplementary Fig. 1).

With regard to differential expression analysis, 1027 ERGs were obtained from an EMT gene database, “dbEMT2” [26], and DEERGs between the LN-positive and LN-negative groups were screened by the ‘limma’ package [27]. A false discovery rate (FDR) < 0.05 and |logFC|> 1 were defined as the significance threshold. On the basis of DEERGs, Gene Ontology (GO) and Kyoto Encyclopedia of Genes and Genomes (KEGG) pathway analyses were carried out. The STRING database and Cytoscape were adopted to construct a protein–protein interaction (PPI) network to visualize the relationships among DEERGs, where 0.4 was defined as the minimum required interaction score, and genes with node degree > 15 were considered as hub genes [28–30].

For the sake of effectively screening candidate genes to construct a risk score for LNM, we adopted the “pROC” package to evaluate their predictive value [31]. An area under the curve (AUC) > 0.7 was considered acceptable for further evaluation. The logistic regression model was built to evaluate the predictive value of the genes screened, on the basis of which a nomogram was plotted. According to the probability predicted by the model, sensitivity, specificity, positive predictive value (PPV), and negative predictive value (NPV) were reported. The consistency between the observed incidence rate and the predicted probability of the nomogram was evaluated by adopting a calibration plot with 2000 bootstrap replications. Meanwhile, the Hosmer and Lemeshow tests were used. We also introduced an EC database composed of 95 patients from the Clinical Proteomic Tumor Analysis Consortium (CPTAC) for external validation [32].

All the analyses were conducted by means of R software (version 4.1.1) [33]. Apart from the statistical methods above, the Wilcoxon test and chi-square test were adopted to compare continuous and categorical variables, respectively. A *P* < 0.05 was considered statistically significant.

## Results

A total of 498 patients diagnosed between 1995 and 2013 were included in this study (Table 1), with 15.1% of patients exhibiting LNM. Tumor grade, histological subtype, myometrial invasion (MI), and molecular subtype were significantly associated with LNM (*P* < 0.001).

**Table 1:** Summary of the clinicopathological features of the patients stratified by LN status

Variables	LN-negative (*n* = 423)	LN-positive (*n* = 75)
Age (years), mean ± SD	63.8 ± 11.1	63.3 ± 10.4
Race, *n* (%)		
White	294 (69.5%)	45 (60.0%)
Black or African American	78 (18.4%)	21 (28.0%)
Asian	17 (4.0%)	2 (2.7%)
Others	8 (1.9%)	3 (4.0%)
Tumor grade, *n* (%)		
Grade1	95 (22.5%)	2 (2.7%)
Grade2	109 (25.8%)	8 (10.7%)
Grade3	219 (51.8%)	65 (86.7%)
Histological subtype, *n* (%)		
Endometrioid	350 (82.7%)	35 (46.7%)
Non-endometrioid	73 (17.3%)	40 (53.3%)
MI, *n* (%)		
< 1/2	285 (67.4%)	26 (34.7%)
≥ 1/2	138 (32.6%)	49 (65.3%)
Molecular subtype, *n* (%)		
*POLE*	39 (9.2%)	8 (10.7%)
MSI-H	134 (31.7%)	14 (18.7%)
CNL	139 (32.9%)	10 (13.3%)
CNH	103 (24.3%)	42 (56.0%)
Missing	8 (1.9%)	1 (1.3%)

*Abbreviations*: *LN* Lymph node, *SD* Standard deviation, *EEC* Endometrioid endometrial cancer, *MI* Myometrial invasion, *MSI-H* Microsatellite instability-high, *CNL* Copy-number low, *CNH* Copy-number high

### EMT scores stratified by LN status

A total of 314 ERGs (144 epithelial and 170 mesenchymal) were included to evaluate the EMT status, with a pseudogene *OR7E14P* excluded on account of its absence in the TCGA database.

**Fig. 1:**
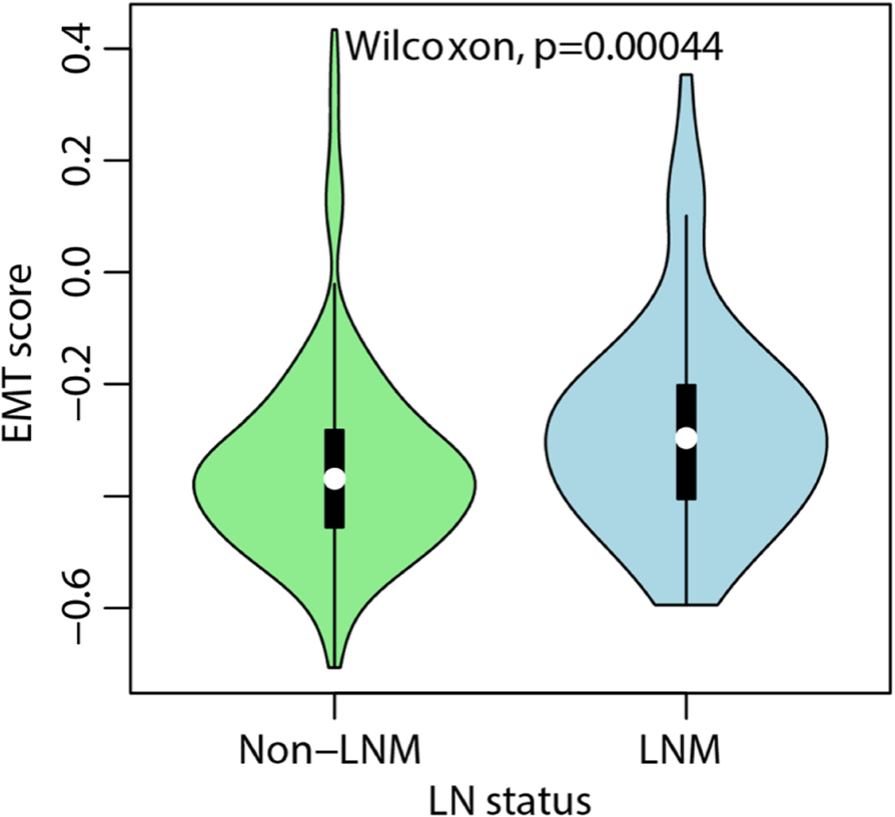
EC tissues from LNM cases exhibited a more mesenchymal phenotype compared with their non-LNM counterparts

Coinciding with the epithelial origin of EC, general expressions of ERGs revealed a more epithelial phenotype [median − 0.361, IQR (− 0.449, − 0.264)] in the whole cohort. The median EMT score of the LN-negative group was − 0.369 [IQR (− 0.455, − 0.282)], while that of the LN-positive group was − 0.296 [IQR (− 0.449, − 264)], with a *P* < 0.001 by Wilcoxon test, which presented a more mesenchymal phenotype for tumors with LNM on the EMT continuum (Fig. 1). Further subgroup analysis revealed that this phenotype difference was more significant in G3, MI ≥ 1/2, and *POLE* cases (Supplementary Fig. 2).

### Differentially expressed genes and pathway analysis

By comparing mRNA expression profiles of tumor tissues from 423 LN-negative cases and 75 LN-positive cases, 266 genes were identified as DEERGs, in which 184 were upregulated and 82 were downregulated. Expressions of ERGs were exhibited in Supplementary Fig. 3 and Supplementary Table 1.

**Fig. 2:**
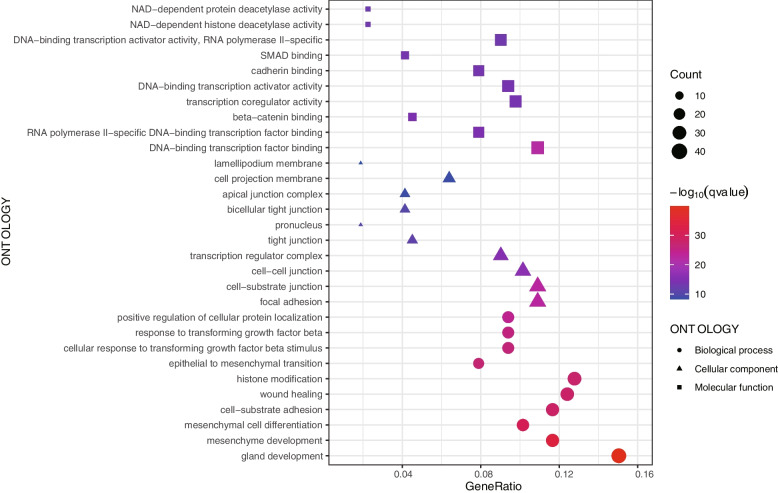
Various EMT-related pathways were enriched, including the TGF-β signaling pathway. EMT, epithelial-mesenchymal transition; TGF-β, transforming growth factor-beta

GO enrichment terms on the basis of DEERGs were exhibited in Fig. 2. In the biological process (BP) group, various EMT-related processes were enriched (Supplementary Table 2), e.g., mesenchyme development (*q* = 1.49 × 10^−15^), mesenchymal cell differentiation (*q* = 3.02 × 10^−14^), epithelial to mesenchymal transition (*q* = 1.97 × 10^−12^), cellular response to transforming growth factor beta stimulus (*q* = 4.14 × 10^−12^), response to transforming growth factor beta (*q* = 6.37 × 10^−12^), exhibiting a propensity for the enrichment of TGF-β signaling pathway. A directed acyclic graph (DAG) displaying the correlation between the BPs enriched was as Supplementary Fig. 4.

**Fig. 3:**
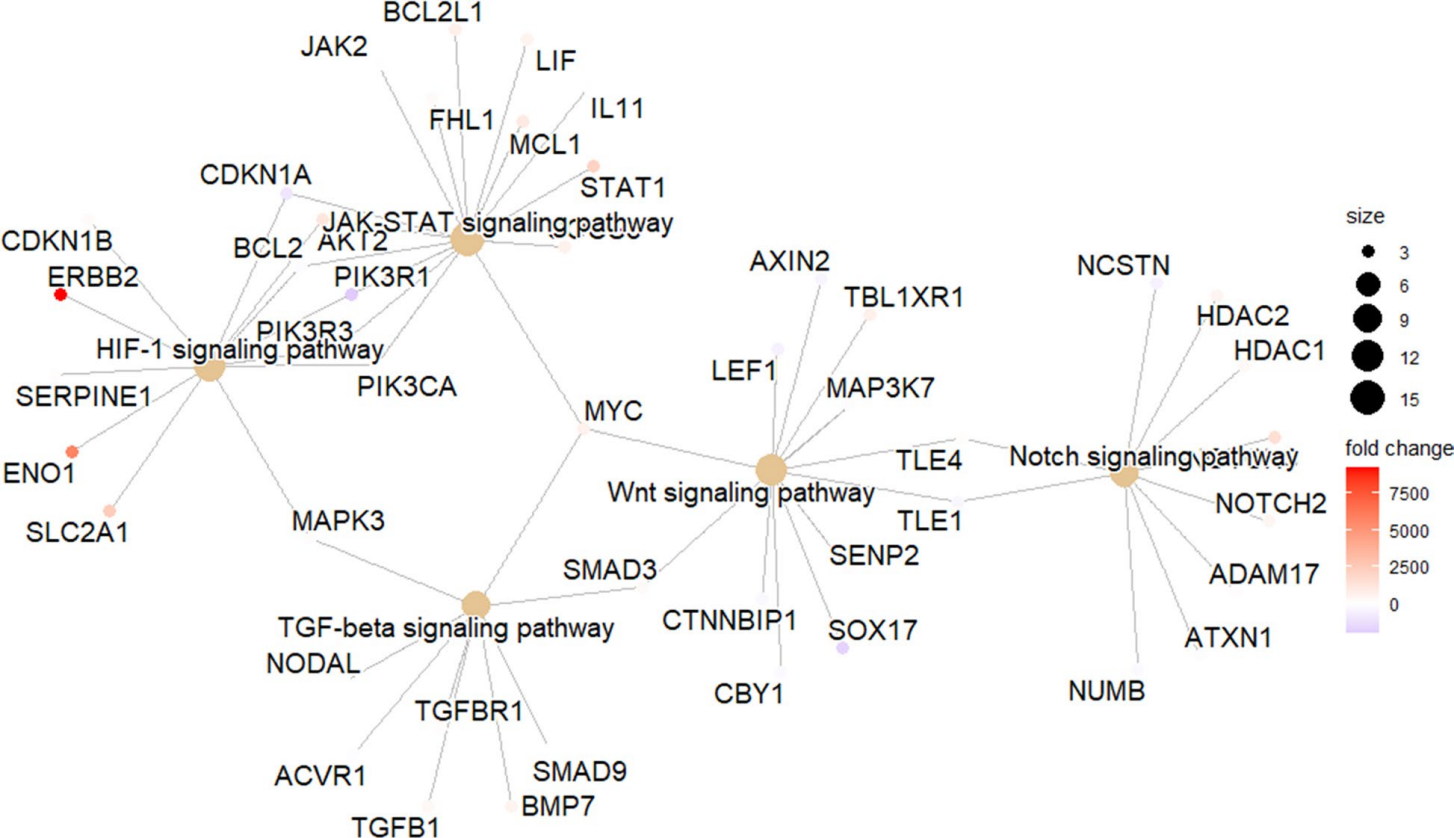
Several EMT-related pathways enriched by KEGG. EMT, epithelial-mesenchymal transition; KEGG, Kyoto Encyclopedia of Genes and Genomes

KEGG pathway analysis exhibited a significant enrichment in EMT-related pathways, e.g., Notch signaling pathway, JAK-STAT signaling pathway, HIF-1 signaling pathway, TGF-β signaling pathway, and Wnt signaling pathway, most members of which exhibited an upregulation in LNM cases (Fig. 3, Supplementary Table 3).

The PPI network of DEERGs based upon the STRING database was presented in Supplementary Fig. 5. The top 5 hub genes calculated by Edge Percolated Component (EPC) method were *MYC*, *ESR1*, *KRAS*, *MAPK3*, and *HDAC1*. We also summarized hub genes by each method in Supplementary Table 4.

**Fig. 4:**
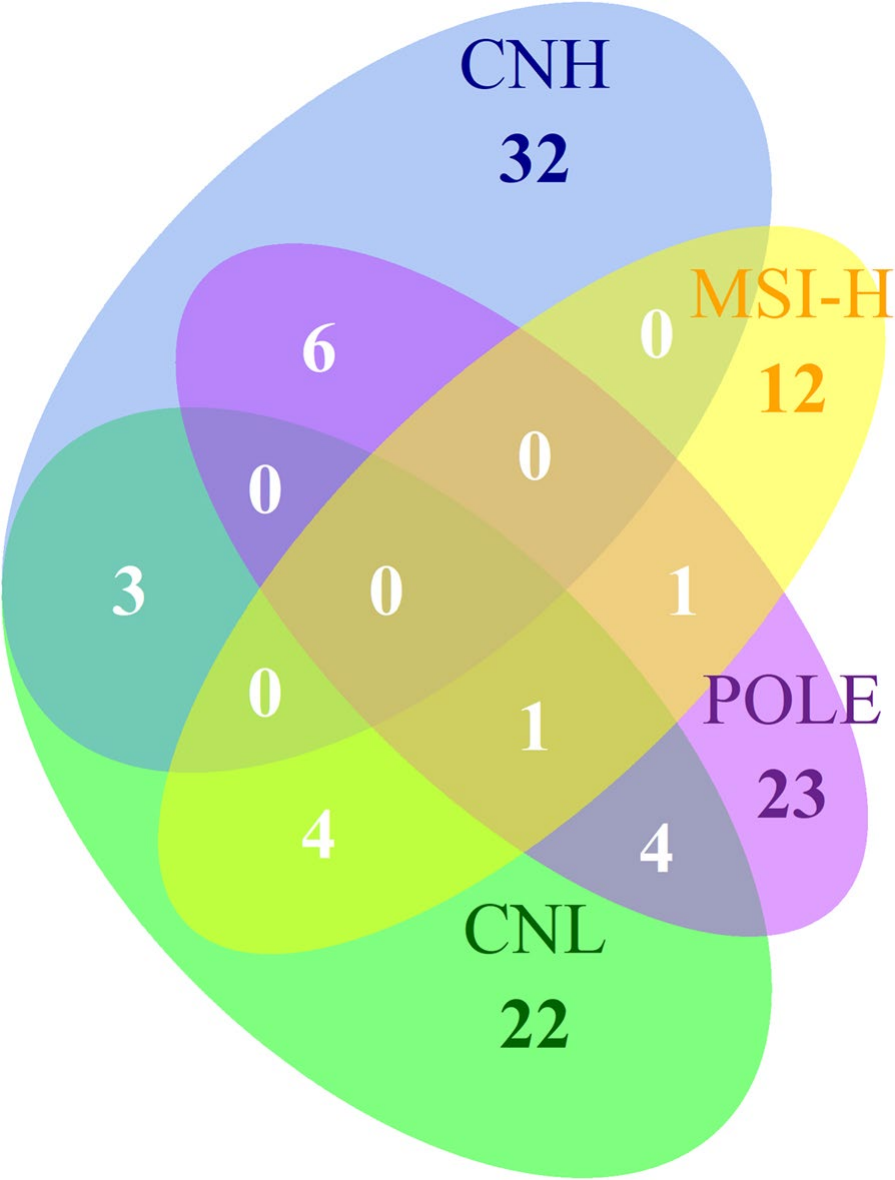
DEERGs shared between molecular subtypes were comparatively few. DEERG: differentially expressed EMT-related genes

As is exhibited in Table 1, molecular subtypes of 96.8% of patients were available, and CNH cases were more likely to develop LNM than the others (*χ*^2^ = 30.5, *P* < 0.001). Therefore, we investigated DEERGs associated with LNM in cases with molecular subtypes (*n* = 491, Supplementary Table 5). A Venn graph displaying DEERGs shared between subtypes was depicted in Fig. 4, where DEERGs differed tremendously between subtypes. No DEERGs were shared between all subtypes, and only *HOXB7*, a gene encoding an activator of the TGF-β pathway, was shared by CNL, MSI-H, and *POLE*.

### Identification of a gene signature effective to predict LNM

**Fig. 5:**
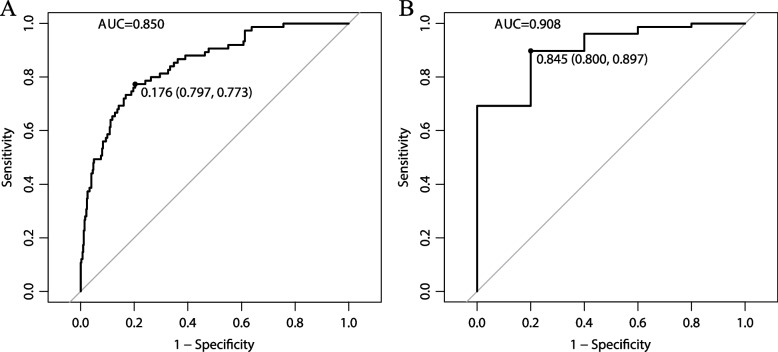
A logistic regression model combining expression-based risk score and clinicopathological parameters exhibited a good discrimination in the TCGA (**A**) and CPTAC (**B**) dataset

On the basis of receiver operating characteristic (ROC) curves adopted to discriminate between LN-positive and LN-negative groups, no DEERG exhibited an AUC > 0.7 (Supplementary Table 6). Consequently, a logistics regression model was constructed to screen candidate genes by including DEERG expression, age, race, grade, MI, and histological subtype, ROC curves from which were employed to screen target genes. As a result, the median AUC was 0.802 (IQR 0.801–0.805), which displays a comparatively high predictive value of the new models. To refine the candidate genes, genes from models with an AUC > 0.7 and *P* < 0.05 were selected for further analysis. Subsequently, 29 genes were selected (Supplementary Table 7), which were included in a further logistic regression model, and ultimately 7 genes with the best performance were selected: *CIRBP*, *DDR1*, *F2RL2*, *HOXA10*, *PPARGC1A*, *SEMA3E*, *TGFB1* (Supplementary Table 8). An EMT-based risk score (ERS) was calculated as the logit from the logistic regression as follows: 1.845 + (0.323 × CIRBP − 0.096 × DDR1 − 1.381 × F2RL2 − 0.190 × HOXA10 − 2.348 × PPARGC1A + 1.137 × SEMA3E − 0.569 × TGFB1) × 10^−3^. Then we constructed a new logistic model to predict LNM with ERS, race, age, grade, MI, and histological subtype. A further stepwise algorithm filtered out race, eventually reaching an AUC of 0.850 (Fig. 5A). The cut-off value of the probability predicted by the logistic model is 0.176, with a sensitivity of 0.773, a specificity of 0.797, a PPV of 0.403, and an NPV of 0.952.

**Fig. 6:**
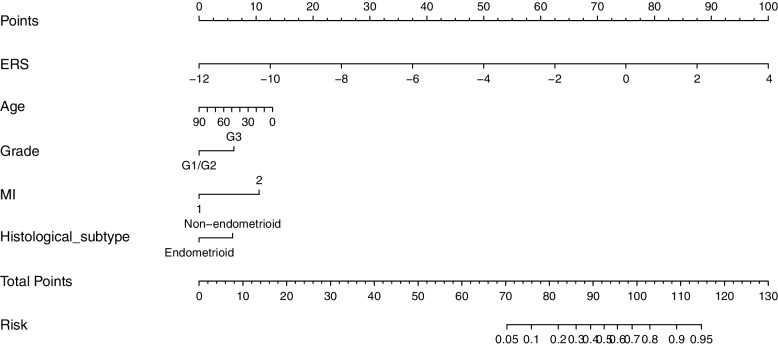
ERS was the primary predictor of LNM in the nomogram

A nomogram based upon the above model was presented in Fig. 6, with ERS having the highest weight in the model compared with traditional clinicopathological parameters. A Hosmer–Lemeshow test exhibited a desirable calibration for this model (*χ*^2^ = 14.3, *P* = 0.074), with a calibration curve in Supplementary Fig. 6.

In an effort to validate the gene signature externally, we extracted mRNA expression profile of EC tissue from the CPTAC program, from which 83 cases identified as stage IA-IIIC2 were included. A similar logistic regression model was built (Supplementary Table 8), with an AUC of 0.908 (Fig. 5B) and an ideal calibration (*χ*^2^ = 3.9, *P* = 0.42). Aside from that, proteome from CPTAC exhibited a significantly positive correlation between proteins and mRNA in members of the 7-gene signature (Supplementary Fig. 7). Nevertheless, owing to the absence of F2RL2 and PPARGC1A proteins, the value of a protein-based ERS was not assessed in this study.

## Discussion

LNM had an incidence of 18.1% in EC, which was quite difficult to predict in clinical practice [3]. In our institution, EC patients with MI ≤ 1/2, G1/2, and endometrioid histology, with no further evidence of invasion/metastasis, used to undergo a surgery without pelvic and para-aortic lymphadenectomy, all the others would receive lymphadenectomy, with sentinel lymphadenectomy (SLND) optional for patients of relatively low risk. Nevertheless, non-therapeutic systemic lymphadenectomy still existed in most cases (71.4%, unpublished data), which gives rise to an augment in the complication morbidity, e.g., blood loss, lymphatic cyst, and lower limb edema [34]. Meanwhile, variables employed to determine lymphadenectomy were mostly those significantly correlated with LNM. The molecular subtype has been proven to be significantly correlated with LNM [3], but has not been extensively adopted in the decision of lymphadenectomy yet. At the mechanism level, LNM is a complex biological process where EMT plays a central role in the invasion of lymphatic vessels and survival in LNs [35, 36], literature concentrating on the role of EMT in LNM in EC remains few [18]. On that account, our team designed this first TCGA-based study combining transcriptomic data with conventional parameters to explore EMT-related molecular characteristics of LNM in EC.

We applied a well-validated EMT quantification method to describe a tumor sample on the EMT continuum [25]. Though unable to discriminate hybrid E/M samples from a mixture of E&M ones, this model strongly suggested samples from LNM cases tended to have a more mesenchymal phenotype, consistent with the association between EMT and LNM in the literature, which exhibited the feasibility of further exploration of this process in EC. Intriguingly, this difference in EMT score was more significant in G3 and MI ≥ 1/2 cases, which indicates an association between invasive phenotypes and the role of EMT in LNM. In all the molecular subtypes, LNM cases tended to have higher EMT scores, with *POLE* showing statistical significance (Supplementary Fig. 2). This indicated a possible variation between molecular subtypes regarding the extent of dependence on EMT during LNM. There is a blank of study on EMT in different molecular subtypes, which seems promising when taking into account the abundant pathways and molecules in the EMT process [37].

Among the 1027 ERGs included in this study, over 1/4 showed a differential expression between LNM and non-LNM cases, with multiple pathways being enriched, in which the TGF-β signaling pathway has been proven a potent inducer of EMT in EC [38]. Nonetheless, no published literature discussed the role of the TGF-β pathway for LNM in EC. In the GO analysis based upon DEERGs, various pathways bound up with TGF-β were enriched in the BP group. A similar result was reached by KEGG analysis, with other EMT-related pathways significantly enriched as well, members of which were mostly upregulated (Fig. 3). Hub genes suggested by PPI analysis, e.g., *Myc*, *ESR1*, and *KRAS*, were reported to participate in EMT pathways, i.e., Wnt, TGF-β, and MAPK signaling pathways [39–44], and they also showed most interactions with other DEERGs. These hub genes might be associated with the core mechanism and containment strategy of LNM in EC in need of further exploration.

Data from this study showed different LNM rates between molecular subtypes, especially for a significantly higher rate in CNH cases, consistent with the report by Jamieson et al. [3]. DEERGs shared between subtypes were comparatively few, which exhibits possibly different landscapes of LNM between molecular subtypes in EC, as stated above. Detailed pathway analyses were omitted as a result of a small sample size in the subgroup analysis, and a prospective study on EMT in LNM and its correlation with molecular subtype in EC is being carried out in our institution (NSFC81972426).

As mentioned earlier, an accurate method of calculating the LNM probability will contribute to a precise strategy for lymphadenectomy and adjuvant therapy. Several predictive models with desirable performance have been created, predominantly based upon clinicopathological parameters [45, 46]. But before the definitive surgery, molecular features of EC can be acquired through hysteroscopy samples as well [47], providing a chance to calculate the risk of LNM more comprehensively. With the logistic regression model, we ultimately reached a gene signature composed of 7 DEERGs, the new model based upon which exhibited a satisfactory discrimination and calibration. It is also pivotal to note that in accordance with the nomogram, ERS accounted for the majority of LNM risk, which demonstrated that molecular features from the primary tumor did provide significant information of LNM, which is independent from traditional clinicopathological parameters. This partly explained the difficulty to predict LNM in clinical practice, for traditional models (e.g., Mayo criteria) lacked paramount molecular features. In the TCGA-based logistic model, a PPV of 0.403 and an NPV of 0.952 were reached, which seems acceptable owing to the comparatively uncommon and unpredictable nature of LNM in EC. In terms of screening candidates for abandoning lymphadenectomy, a model with an NPV high enough is more crucial for medical safety [48, 49]. What is more, it is worth mentioning that SLN detection has been proven to accurately detect LNM in EC [50, 51], effectively lessen complications associated with systemic lymphadenectomy. For the time being, the number of research literature on molecular characteristics guiding SLN detection applications is still limited (predominantly molecular subtypes) [52], which evidently exhibits a promising prospect for molecular feature-guided lymphadenectomy.

The value of the 7-gene signature was further validated in a new EC database from CPTAC, where most members of the signature exhibited a positive correlation between proteins and mRNA, which conspicuously illustrates that to transform this transcriptome-based gene signature into an immunohistochemistry-based tissue microarray (TMA) from hysteroscopy biopsies is promising in clinical practice. In accordance with the latest report of Jamieson et al. on molecular subtype and LNM in EC, the molecular subtype is a potential predictor of LNM [3]. And the 7 genes proposed in this study are not covered by the commonly used Trans-PORTEC and ProMisE algorithms, i.e., the 7-gene signature might provide independent predictive value preoperatively on the basis of molecular subtype. Moreover, different molecular subtypes of EC had different DEERGs in LNM (Fig. 4), which suggested the existence of different EMT pathways and variant dependence on EMT during the lymphatic invasion in EC. When EMT inhibitors are applied into clinical research to control metastasis in the future (as in anti-PD-1/PD-L1) [53], this study may contribute to precise medication in line with molecular features in EC.

There are several limitations in our research. First, owing to the heterogeneity of transcriptome from different databases, our study did not establish a universally applicable clinical prediction model. But the ERG-based gene signature did exhibit a predictive value independent of clinicopathological parameters across databases, supporting future clinical applications. Aside from that, this study is simply limited to bioinformatics analysis, not verified by specimens from our institution yet. Furthermore, the above limitations can be remedied through developing a TMA with a unified standard. Meanwhile, there is a huge gap for laboratory research to fill on the mechanisms of EMT to induce lymphatic spread in EC.

## Conclusions

To sum up, our study suggested the EMT status and expression of ERGs varied in LNM and non-LNM EC tissues, involving multiple EMT-related signaling pathways, and the distribution of DEERGs differed among molecular subtypes. Furthermore, an ERG-based gene signature including 7 DEERGs exhibited a desirable predictive value for LNM in EC, which requires further validation on the basis of clinical specimens in the future.

## Supplementary Information

**Additional file 1: Supplementary figure 1.** A diagram of the mechanism of the KS method, exhibiting a tumor tissue with a more mesenchymal phenotype. The lower ECDF curve represented the higher expression of mesenchymal gene signatures, thus the EMT score of the presented sample is positive. **Supplementary figure 2.** EMT scores were distributed unevenly when stratified by grade (A), histological subtype (B), MI (C) and molecular subtypes (D). LN: lymph node; MI: myometrial invasion; CNH: Copy-number high; CNL: copy-number low; MSI-H: microsatellite instability-high. **Supplementary figure 3.** Heatmap for ERGs between tumor samples from LN positive and negative cases (normalized for exhibition). ERG: EMT-related gene; LN: lymph node. **Supplementary figure 4.** A DAG displaying the relationship between BPs from GO analysis. DAG: directed acyclic graph. BP: biological process; GO: Gene Ontology. **Supplementary figure 5.** PPI network by Cytohubba, with top 5 hub genes highlighted by colors. PPI: protein–protein interaction. **Supplementary figure 6.** Calibration plot suggesting predicted probabilities of LNM corresponded closely to the actually observed proportions. LNM: lymph node metastasis. **Supplementary figure 7.** A significantly positive correlation between proteins and mRNA in members of the 7-gene signature based on CPTAC. X axis: mRNA, Y axis: protein. CPTAC: Clinical Proteomic Tumor Analysis Consortium.

**Additional file 2: Supplementary table 1.** DEERGs between LN positive and negative cases were screeened out by 'limma' package.

**Additional file 3: Supplementary table 2.** Gene Ontology (GO) analyses were carried out by 'enrichplot' package.

**Additional file 4: Supplementary table 3.** Kyoto Encyclopedia of Genes and Genomes (KEGG) analyses were carried out by 'enrichplot' package.

**Additional file 5: Supplementary table 4.** Top 5 hub genes calculated by each method in PPI analysis.

**Additional file 6: Supplementary table 5.** DEERGs and their predictive value in LNM in cases with molecular subtypes.

**Additional file 7: Supplementary table 6.** DEERGs and their predictive value between LN positive and negative cases.

**Additional file 8: Supplementary table 7.** DEERGs and their predictive value in LNM when combined with clinicopathological parameters.

**Additional file 9: Supplementary table 8.** The 7 genes with the best performance in logistic regression were selected, with ERS based on which, both in TCGA and CPTAC database.

## Data Availability

All data of this research are from public databases, and algorithms and annotated data in this research can be requested from the corresponding author on the basis of academic collaboration.

## Abbreviations

AUC: Area under the curve
BP: Biological process
CNH: Copy-number high
CNL: Copy-number low
CPTAC: Clinical Proteomic Tumor Analysis Consortium
DAG: Directed acyclic graph
DEERG: Differentially expressed ERG
EC: Endometrial cancer
ECDF: Empirical distribution function
EEC: Endometrioid endometrial cancer
EMT: Epithelial-mesenchymal transition
EPC: Edge Percolated Component
ERG: EMT-related gene
ERS: EMT-based risk score
GO: Gene Ontology
IQR: Interquartile range
KEGG: Kyoto Encyclopedia of Genes and Genomes
LNM: Lymph node metastasis
MI: Myometrial invasion
MSI-H: Microsatellite instability-high
NPV: Negative predictive value
PPI: Protein–protein interaction
PPV: Positive predictive value
ROC: Receiver operating characteristic
SD: Standard deviation
SLND: Sentinel lymphadenectomy
TCGA: The Cancer Genome Atlas
TGF-β: Transforming growth factor-beta
TMA: Tissue microarray
