# Correction: Expression of EMT-related genes in lymph node metastasis in endometrial cancer: a TCGA-based study

**DOI:** 10.1186/s12957-023-02979-x

**Published:** 2023-03-14

**Authors:** He Li, Junzhu Wang, Liwei Li, Luyang Zhao, Zhiqi Wang

**Affiliations:** 1 Department of Obstetrics and Gynecology, Peking University People’s Hospital, No. 11 Xizhimen South Street, Xicheng District, Beijing, 100044 China; 2 The Big Data and Public Policy Laboratory, School of Government, Peking University, Beijing, China

**Correction: World J Surg Oncol 21, 55 (2023)**

**https://doi.org/10.1186/s12957-023-02893-2**

Following publication of the original article [1], the authors identified an error in the author names of all authors. The given name and family name were erroneously transposed.

The incorrect author names are:

Li He, Wang Junzhu, Li Liwei, Zhao Luyang and Wang Zhiqi

The correct author names are:

He Li, Junzhu Wang, Liwei Li, Luyang Zhao and Zhiqi Wang

The author group has been updated above and the original article [1] has been corrected.

Example
