# Supplementary material for: Expression of EMT-related genes in lymph node metastasis in endometrial cancer: a TCGA-based study
Source: World J Surg Oncol. 2023 Feb 22;21:55. doi: 10.1186/s12957-023-02893-2 (PMC9945723; doi:10.1186/s12957-023-02893-2)
Supplement: Supplementary file 1 — Additional file 1: Supplementary figure 1. A diagram of the mechanism of the KS method, exhibiting a tumor tissue with a more mesenchymal phenotype. The lower ECDF curve represented the higher expression of mesenchymal gene signatures, thus the EMT score of the presented sample is positive. Supplementary figure 2. EMT scores were distributed unevenly when stratified by grade (A), histological subtype (B), MI (C) and molecular subtypes (D). LN: lymph node; MI: myometrial invasion; CNH: Copy-number high; CNL: copy-number low; MSI-H: microsatellite instability-high. Supplementary figure 3. Heatmap for ERGs between tumor samples from LN positive and negative cases (normalized for exhibition). ERG: EMT-related gene; LN: lymph node. Supplementary figure 4. A DAG displaying the relationship between BPs from GO analysis. DAG: directed acyclic graph. BP: biological process; GO: Gene Ontology. Supplementary figure 5. PPI network by Cytohubba, with top 5 hub genes highlighted by colors. PPI: protein–protein interaction. Supplementary figure 6. Calibration plot suggesting predicted probabilities of LNM corresponded closely to the actually observed proportions. LNM: lymph node metastasis. Supplementary figure 7. A significantly positive correlation between proteins and mRNA in members of the 7-gene signature based on CPTAC. X axis: mRNA, Y axis: protein. CPTAC: Clinical Proteomic Tumor Analysis Consortium. [file 12957_2023_2893_MOESM1_ESM.docx]

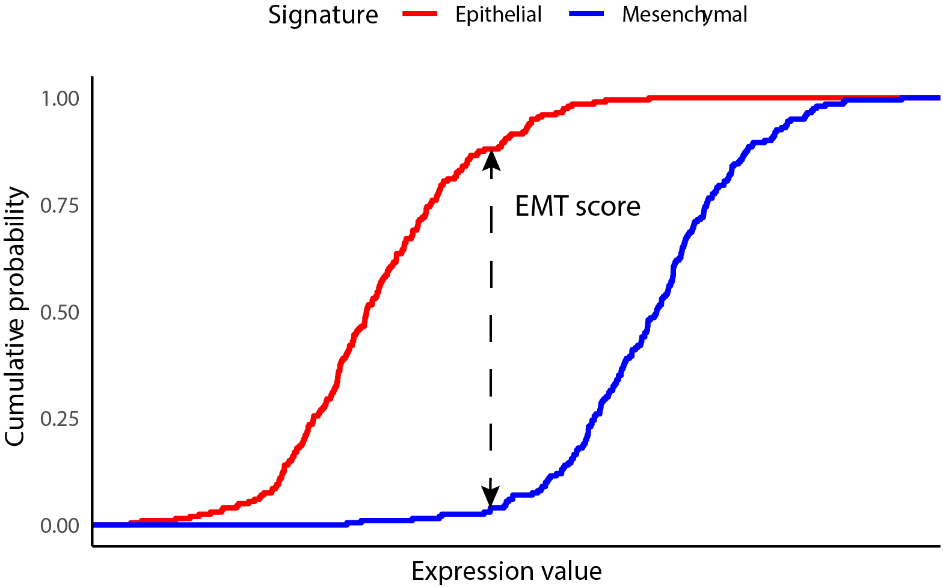


Supplementary figure 1 A diagram of the mechanism of the KS method, exhibiting a tumor tissue with a more mesenchymal phenotype. The lower ECDF curve represented the higher expression of mesenchymal gene signatures, thus the EMT score of the presented sample is positive.


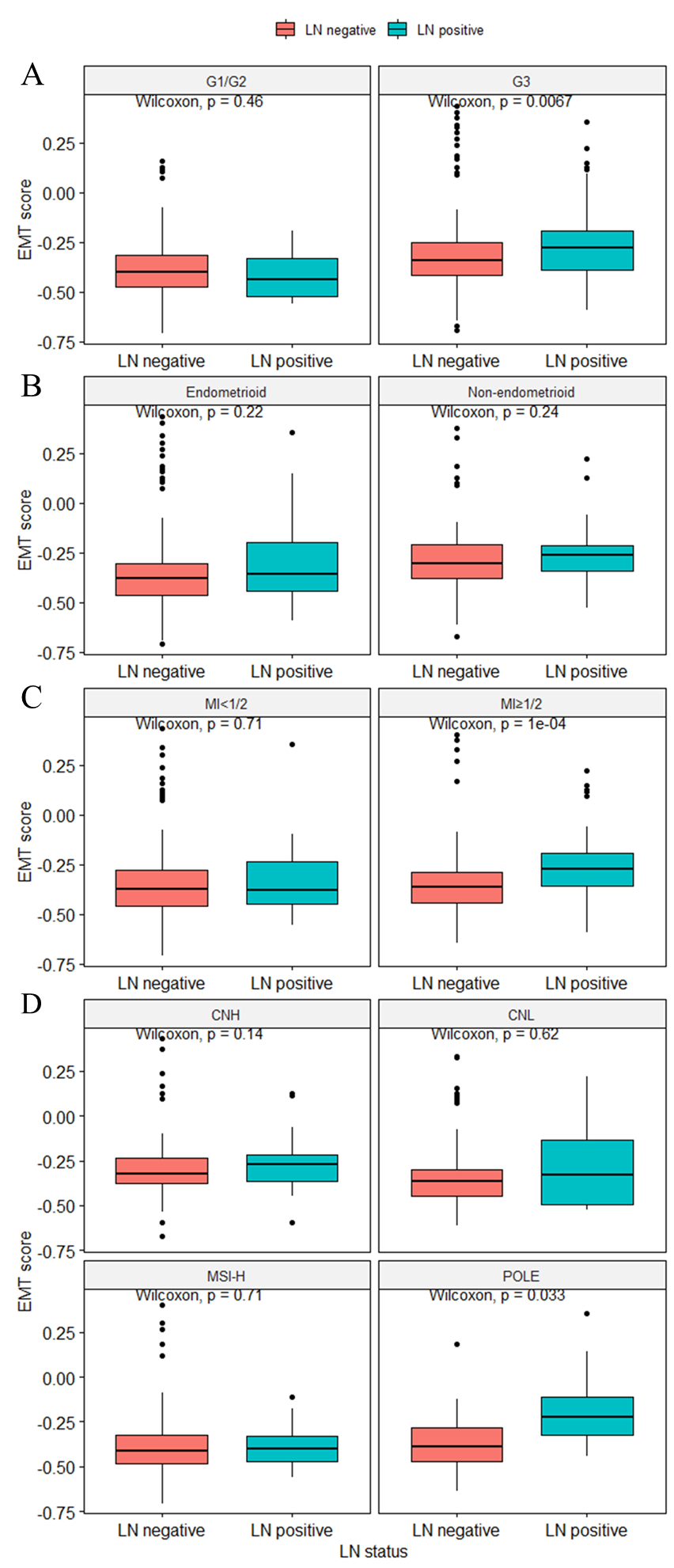


Supplementary figure 2 EMT scores were distributed unevenly when stratified by grade (A), histological subtype (B), MI (C) and molecular subtypes (D). LN: lymph node; MI: myometrial invasion; CNH: Copy-number high; CNL: copy-number low; MSI-H: microsatellite instability-high.


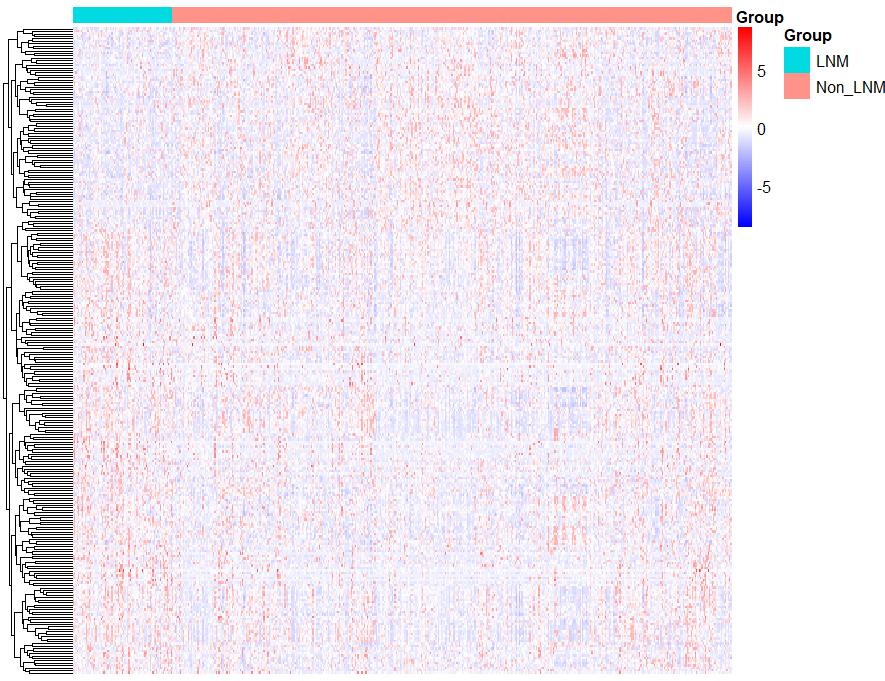


Supplementary figure 3 Heatmap for ERGs between tumor samples from LN positive and negative cases (normalized for exhibition). ERG: EMT-related gene; LN: lymph node.


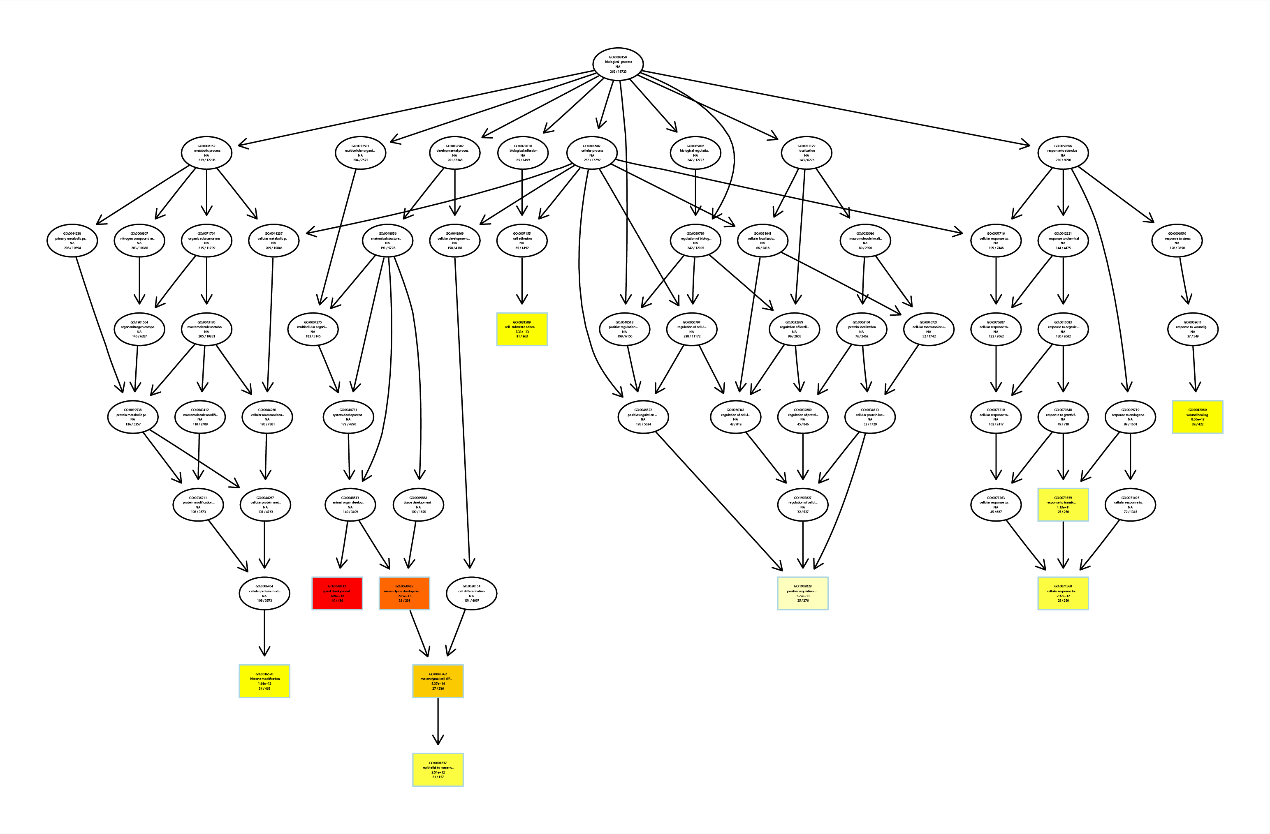


Supplementary figure 4 A DAG displaying the relationship between BPs from GO analysis. DAG: directed acyclic graph. BP: biological process; GO: Gene Ontology.


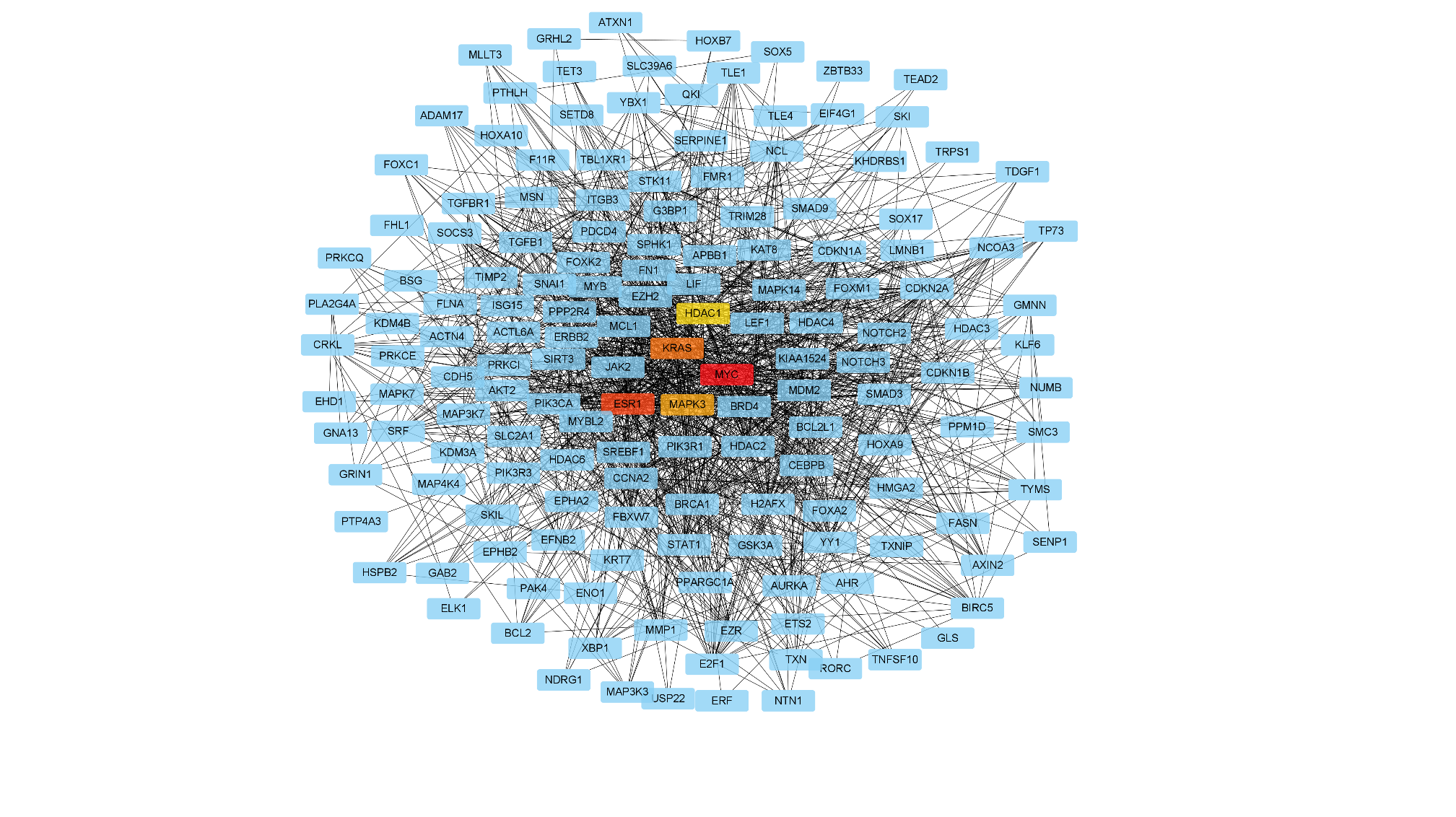


Supplementary figure 5 PPI network by Cytohubba, with top 5 hub genes highlighted by colors. PPI: protein–protein interaction.


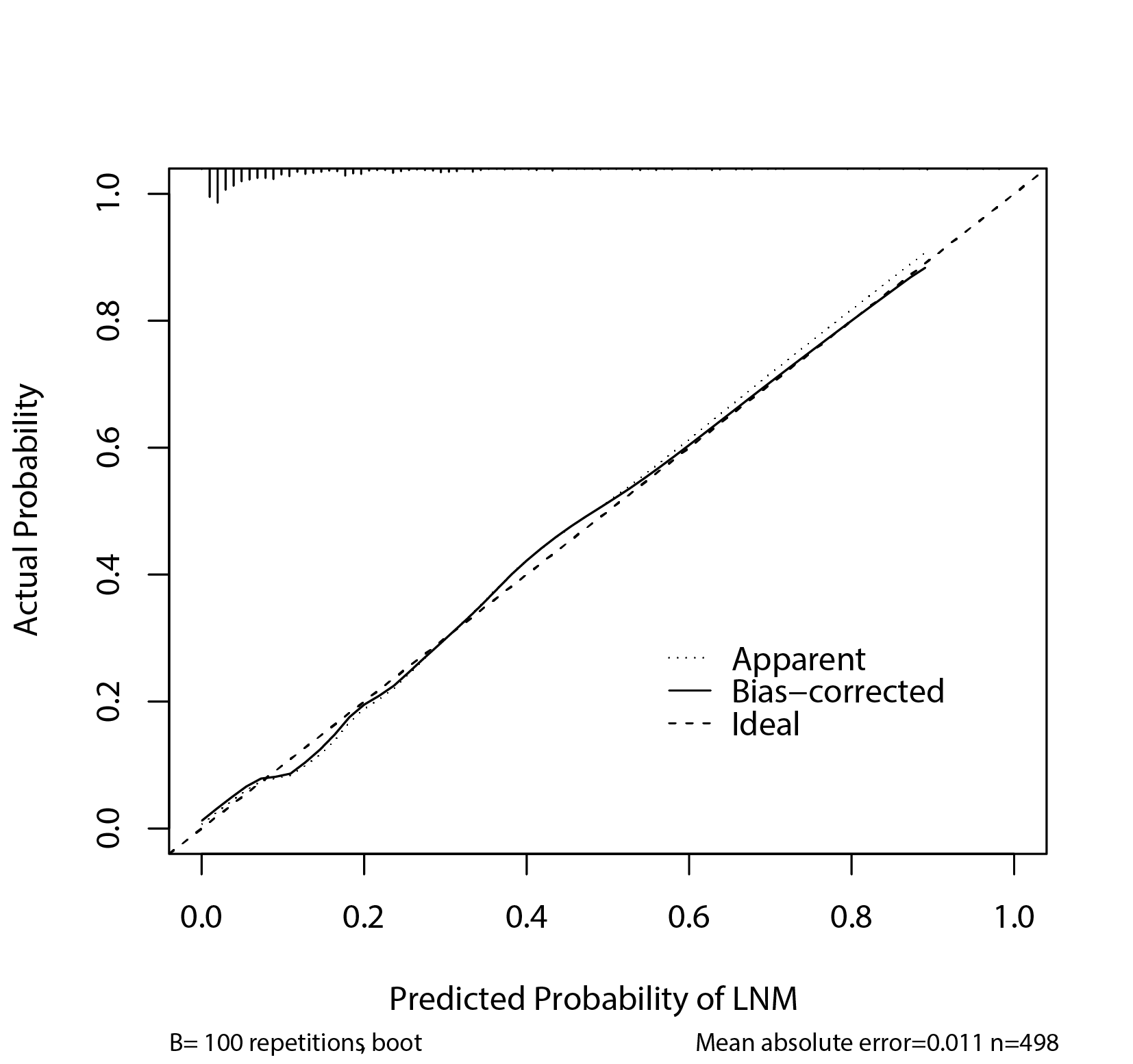


Supplementary figure 6 Calibration plot suggesting predicted probabilities of LNM corresponded closely to the actually observed proportions. LNM: lymph node metastasis.


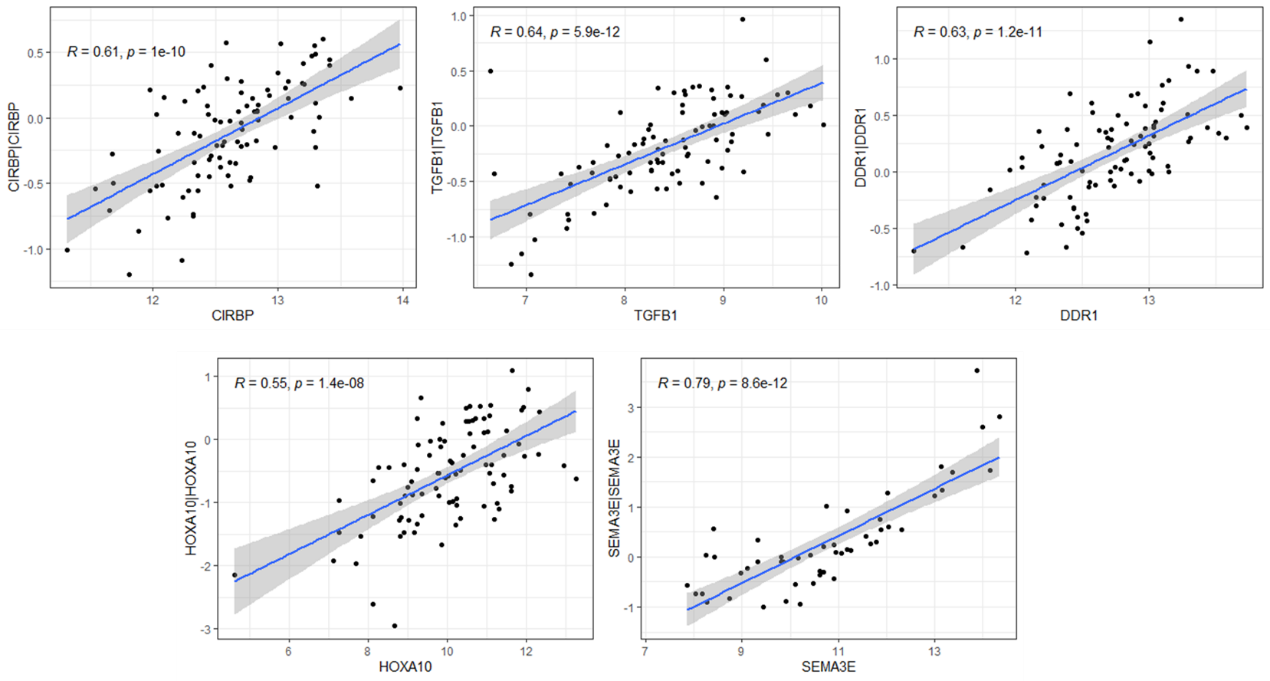


Supplementary figure 7 A significantly positive correlation between proteins and mRNA in members of the 7-gene signature based on CPTAC. X axis: mRNA, Y axis: protein. CPTAC: Clinical Proteomic Tumor Analysis Consortium.
